# Breaking refractive index records with layered van der Waals GeS_2_ for blue and near-ultraviolet photonics

**DOI:** 10.1038/s41377-025-02070-y

**Published:** 2026-01-02

**Authors:** Pavel Shafirin, Mozakkar Hossain, Artur Davoyan

**Affiliations:** Department of Mechanical and Aerospace Engineering, University of California, Los Angeles, 90095 CA USA

**Keywords:** Nanophotonics and plasmonics, Sub-wavelength optics

## Abstract

GeS_2_, a layered a wide bandgap van der Waals material, is now found to exhibit record-high refractive index and extreme optical anisotropy across blue and near-ultraviolet bands, promising bright future for short-wavelength photonics.

Efficient harnessing of blue and near-ultraviolet radiation – spanning the 400–500 nm and 300–400 nm wavelength bands, respectively—is a long standing goal of photonic sciences. A wide range of critical applications lie in this wavelength range from taming emission of ultraviolet and blue LEDs through polymer curing, ink printing, and lithography to third harmonic generation by Nd:YAG lasers, and to forensics and security applications. However, the short wavelengths, and consequently high photon energies, of blue and near-ultraviolet light impose strict constraints on the choice of suitable materials. To avoid optical absorption, materials must possess large optical bandgap (${E}_{g} > 3.5\mathrm{eV}$). While many transparent insulators and wide-bandgap semiconductors exist, including, among others, LiNbO_3_, TiO_2_, Al_x_Ga_1-x_N, Si_3_N_4_ and hBN, their large bandgap inherently translates to a relatively small index of refraction, *n*. An inverse relation between materials bandgap and their refractive index, is well captured by an empirical Moss rule^1^: $n=\root{{4}}\of{95\,eV/{E}_{g}}$. As a result, unlike a plethora of high-index semiconductors suitable for red-light and infrared bands, blue-light and near-ultraviolet materials exhibit refractive indices typically limited to *n* < 2.5, Fig. 1 (left).

**Fig. 1:**
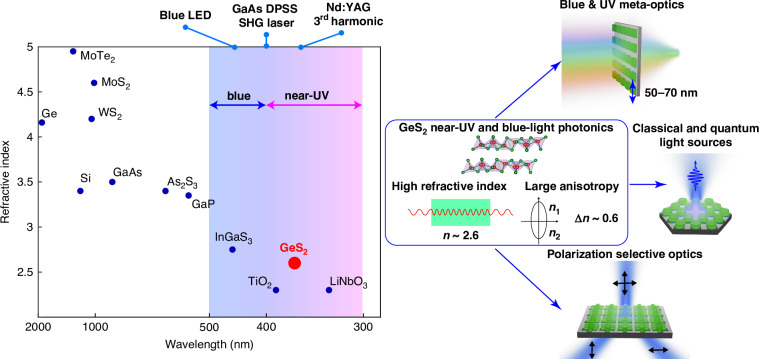
Germanium disulfide for near-ultraviolet and blue-light photonics. (left) Refractive index versus optical bandgap wavelength for several typical photonics materials. Shaded areas denote blue and near-ultraviolet parts of the spectrum. Select technologically relevant wavelengths are highlighted. (right) Schematic illustration of several of near-ultraviolet and blue-light applications, where GeS_2_’s high refractive index and large anisotropy could yield a transformative leap

Refractive index, on the other hand, governs light-materials interaction and sets constraints on the overall size and efficiency of photonic devices. Materials with a higher index of refraction imply shorter effective wavelength, smaller phase and group velocities. As such, devices made of high-index materials exhibit smaller size features (typically scaled as *λ/*2*n*, where *λ* is a free space wavelength) and tighter optical mode confinement – both of which are prerequisites for efficient quantum and classical light manipulation. Recently, a number of works have shown that van der Waals materials owing to their unique electronic structure, stemming from their layered nature, *d*-shell valence electrons, and sharp room temperature excitonic resonances, are uniquely suited to break refractive index records set by traditional covalent materials^2^. Hence, in the near-infrared range ($\lambda > 1\,\mathrm{\mu m}$) electronically bulk transition metal dichalcogenides, mainly Mo and W compounds, demonstrate refractive indices 25–30% higher than that of Si and GaAs, Fig. 1 (left)^3,4^. High index of these materials is of great promise for metasurfaces with deep subwavelength features^5^, high-efficiency nonlinear optics^6,7^, and small-footprint high-density integrated photonics^8,9^, among other infrared applications. At the same time, to date no wide bandgap high-index van der Waals materials have been known for blue and near-ultraviolet parts of the spectrum.

In a newly published paper in *Light: Science & Applications*, A.S. Slavich et al. report discovery of a GeS_2_ as a novel transparent and high index van der Waals material for blue and near-ultraviolet wavelengths^10^. First, equipped with polarization-resolved angle-dependent Raman spectroscopy authors examine crystallographic properties of a layered GeS_2_ crystal. The vibrational spectroscopy, combined with ab initio calculations, reveals that GeS_2_ has a low-symmetry monoclinic crystal structure, which combined with its layered nature, leads to a strong bi-axial anisotropy. Comprehensive spectroscopic ellipsometry on thin mechanically exfoliated films is performed to deduce full tensor structure of optical constants and their wavelength dispersion. Measurements demonstrate that along one of its in-plane crystallographic axes GeS_2_ exhibits record high, for a wide bandgap semiconductor (${E}_{g}\simeq 3.4\,eV-3.7\,eV$; ${\lambda }_{g}\simeq 335\,nm-365\,nm$), refractive index values: Refractive index is in the range $n\simeq 2.5-2.6$ across a large portion of blue and near-ultraviolet parts of the spectrum, reaching $n\simeq 2.8$ close to the onset of optical bandgap at $\simeq 350\mathrm{nm}$. These values are significantly higher than those of other ultraviolet grade materials, such as SiO_2_, TiO_2_ and LiNbO_3_, Fig. 1 (left). Importantly, layered nature combined with a low crystal symmetry lead to an extreme optical anisotropy. Measured in-plane birefringence is $\Delta n\simeq 0.12$, whereas its out of plane birefringence reaches astonishing $\Delta n\simeq 0.63$, which makes GeS_2_ one of the highest known naturally occurring optically anisotropic materials.

A combination of wide bandgap, low optical loss, record-high refractive index, and extreme anisotropy make GeS_2_ uniquely suited for a range of applications across visible, blue, and near-ultraviolet parts of the spectrum. Figure 1 (right) summarizes a few possible opportunities. Hence, GeS_2_ enables photonic nanostructures with sub-50 nm size features, paving the way to efficient near-ultraviolet and visible light phase gradient metasurfaces. Visible light metasurfaces are of especial importance with a surging interest in artificial and virtual reality applications^11^. Efficient near-ultraviolet meta-optics^12^, in turn, is of interest for lithography and spectroscopy, as well as for shaping second or third harmonic generation from diode-pumped GaAs or Nd:YAG lasers, respectively. Small optical mode volume, when combined with extreme anisotropy and high quality factor nanostructures, could further find use in taming directionality and efficiency of quantum and classical light sources, such as boosting rate of spontaneous emission of color centers or controlling of GaN-based LEDs. Extreme record-high anisotropy additionally offers new avenues for ultra-high contrast polarization selective applications.

While GeS_2_’s record-high index and extreme anisotropy are of great interest, further in-depth studies are needed to bring GeS_2_ to practical and industrial use. Among these are growth and scaling to wafer sizes, development of foundry compatible processes, and integration with conventional semiconductors, such as Si. Further studies would clarify limits of optical absorption, explore nonlinear optical properties, and potential electric tuning of refractive index and anisotropy. Nonetheless, the discovery reported by A.S. Slavich et al. opens a new chapter for blue and near-ultraviolet photonics.
